# WHO simulations for youth engagement in global governance in a post-COVID world: Opportunities and challenges of moving conferences online

**DOI:** 10.7189/jogh.11.03070

**Published:** 2021-05-22

**Authors:** Jiaqi Li, Abinayah John, Emmanouil Tasos, Anna-Lucia Koerling, Charlotte Rendina, Ahmed Elshaer, Constance Brunet, Ahmed Magdy, Alexander Lyszkowski, Kim van Daalen

**Affiliations:** 1 University of Cambridge, School of Clinical Medicine, Addenbrooke’s Hospital, Cambridge, UK; 2 Department of Medical Genetics, University of Cambridge, Cambridge, UK; 3 Brighton and Sussex University Hospitals NHS Trust, Brighton, UK; 4 The Royal (Dick) School of Veterinary Studies, University of Edinburgh, Edinburgh, Scotland, UK; 5 Faculty of Medicine, Mansoura University, Mansoura, Egypt; 6 School of the Biological Sciences, University of Cambridge, Cambridge, UK; 7 NHS Lothian, Edinburgh, Scotland, UK; 8 Cardiovascular Epidemiology Unit, Department of Public Health and Primary Care, University of Cambridge, Cambridge, UK

The COVID-19 pandemic has brought about an accelerated learning revolution as schools, universities and organisations rapidly shifted teachings online. This is a large feat that saw geographical and physical barriers uprooted by the use of technology.

In addition to the altered delivery of formal school-based teachings, other modalities of youth education and engagement have also shifted online, including conferences and model simulations. An example is World Health Organisation (WHO) simulations, which have shown great utility in engaging students to actively participate in global health decision making [1,2].

However, a foundational tenet of the effectiveness of these conferences is human interaction, an experience that is inevitably diminished when perceived through a device screen. In this Commentary, we compare the advantages and limitations of in-person vs online conferences using the Cambridge WHO simulations 2019 (in-person) and 2020 (online) as examples.

A key challenge to online conferences is to recapitulate the extensive verbal and non-verbal communication between delegates. Over online platforms, it can be more difficult to perceive cues, with slower responses to signals and delivery of debates. Of note, the World Health Assembly (WHA) 2020 was delivered online due to the pandemic.

Despite the challenges of organising an online conference, we found that it was possible to recapitulate the benefits of previously in-person events. We have accessed participants’ perspectives on their quality of experience using an identical questionnaire in 2019 and 2020, with data pointing towards no difference in the quality of experience between an in-person and online WHO Simulation (Table S1 in the **Online Supplementary Document**).

In addition, the online nature eliminates physical barriers and brings about new opportunities. Apart from preserving the quality of experience, an online conference has the potential of a wider outreach by removing prohibitive barriers such as high ticket and transport costs or acquiring visas, thus increasing overall accessibility to these events **(****Table 1****)** [6]. From participants’ perspective, attending in-person conferences invariably costs more, while most online conferences can be organised free-of-charge or at a relatively very low registration fee. Delegates in areas with inactive WHO simulation circuits can also participate in such conferences when they otherwise would not have a chance to due to prohibitive logistics, costs and travel restrictions. The result of the elimination of such prohibitive barriers was strikingly apparent: in 2019 (in-person conference), CamWHO saw 55 participants coming from 6 countries and 1 continent (Europe); in 2020 (online conference), CamWHO saw 78 participants coming from 32 different countries across 5 continents (Europe, Asia, North America, South America and Africa). The wider outreach was also reflected in guest speakers: In CamWHO 2020, speakers and panellists came from the UK, the USA, South Africa and Australia, whilst in CamWHO 2019 speakers were all based in the UK. Indeed, online conferences can reach a much broader group of participants and speakers, as long as they have a technological device with stable internet access.

**Table 1:** Comparison of key aspects between in-person and online WHO simulations, using CamWHO 2019 (in-person) and CamWHO 2020 (online) as an example

Aspect of comparison		In-person conference	Online conference
**Quality of experience**	*Understanding of WHO and global health*	The overall understanding of WHO and global health across participants improved after attending the conference (n = 37)	The digital nature of the CamWHO 2020 simulation resulted in similar increase in understanding (n = 45) (Table S1 in the **Online Supplementary Document**)
	*Speaking and debating*	Improvement in delegates’ confidence in speaking and debating (n = 37)	The digital nature of the CamWHO 2020 simulation resulted in similar self-reported improvement (n = 45) (Table S1 in the **Online Supplementary Document**)
**Barriers to participation – COST**	*Ticket cost*	High costs.	Significantly lower, potentially free if sufficient sponsorship is obtained by organisers to provide online platform.
		*For example:*	
		CamWHO 2019 £45 early bird, £50 regular;	
		SheffWHO 2019 £45 early bird, £50 regular [3];	Example: SheffWHO 2020 £5 [3]
		LonWHO 2020 £75 [4]	Example: CamWHO 2020 £7
	*Flight and travel cost*	Variable travel costs.	None; geographical barrier eliminated
		For example: estimated cost of flight tickets from Barcelona: £200; flight from Bangkok: £645; train tickets from Liverpool: £130	
	*Accommodation and food costs*	High costs.	No extra costs for food and accommodation; normal costs.
		Significant for accommodation and food, more expensive compared to normal meals at home.	
**Barriers to participation – other logistical aspects**	*Visa application and cost*	UK visa application process is time-intensive, costly (high fees) and often refused, thus causing an additional prohibitive barrier	A visa is not required to attend an in-person conference reducing one of the fundamental barriers to participation.
	*Sponsorship*	Can be time-consuming and not financially accessible for the organising organisation and/or individual participants.	Lack of pressure of having to find sponsors for the event and/or to participate in the event.
	*Other logistical requirements*	Delegates and organisers must find suitable accommodation and rooms to host various components of the programme.	Access to a stable internet connection.
**Outreach and accessibility**	*Diversity and geographical representation of participants*	Most delegates come from high-income countries such as the United Kingdom, Western Europe or North America [2]. CamWHO 2019 saw participants coming from 6 countries and 1 continent (Europe).	Wider, global reach. CamWHO 2020 saw participants coming from 32 countries and 5 continents (Europe, Asia, North America, South America and Africa).
	*Diversity of speakers*	Guest speakers and panellists are limited by their geographical proximity and availability to travel to and attend the conference, as well as budget limitations by organisers to fund transport and accommodation costs.	Guest speakers and panellists potentially invited from all around the world as geographical barrier is eliminated. For example, CamWHO 2020 included speakers from the UK, the US and South Africa.
**Carbon footprint**		Travel to conferences generates an enormous carbon footprint [5]. Also printing of resources, heating of buildings and rooms.	Usage of technological devices eliminates need for conference travels.
**Other challenges**	*Practice of the simulation*	Apart from physical and logistical challenges described earlier, in-person conferences do not experience the limitations imposed by an online platform.	Practical differences from in-person simulation: delegates sending notes through private messaging, blocs gather together in different chats; making it harder for discussion with other delegates during unmoderated caucuses.

**Figure:**
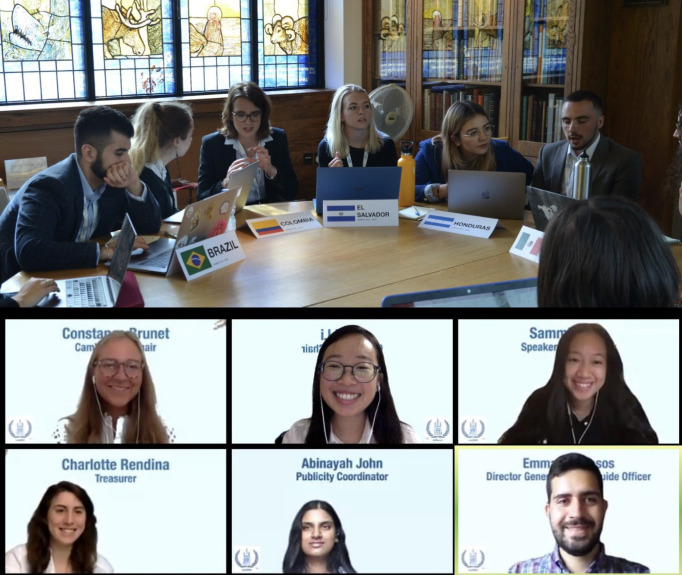
Photo: CamWHO 2019 (in-person) and CamWHO 2020 (online) conferences (from the author’s own collection, used with permission).

Nevertheless, we must acknowledge that access to technological devices and a stable internet connection is not to be taken for granted. A recent report by the World Bank suggests that 65% of the population in low- and middle-income countries do not have internet access [7], and at least 3.6 billion people globally, mainly women, still cannot access the internet, highlighting the digital gender divide [6]. Thus, despite the great promise of narrowing educational inequalities, eConferences such as WHO simulations may contribute and need to consider the widening of the ‘digital divide’ [8].

Ultimately, the purpose of a WHO simulation is to promote youth leadership and engagement in global health governance. Effective global health leadership requires engagement from all perspectives, and discussions should not be dominated solely by wealthy nations. While an online WHO simulation undeniably widens outreach in many regards, the current format still has accessibility limitations, as only participants with access to technological devices and stable internet infrastructure are able to attend. It is thus crucial that future measures are taken to broaden outreach, with the aim of providing opportunities to partake in global health leadership and decision-making to all students with an interest in global health, which is undoubtedly a complex issue to be addressed.

The challenges posed by online simulations are outweighed by the benefits. However, more efforts are needed to address the educational inequalities resulting from the digital divide. Nevertheless, we believe that moving simulations – and conferences alike – online, particularly amidst a ravaging pandemic but also beyond, holds great promise in increasing accessibility, to empower more young individuals around the world in global health leadership.

## Additional material

Online Supplementary Document
