# Supplementary material for: WHO simulations for youth engagement in global governance in a post-COVID world: Opportunities and challenges of moving conferences online
Source: J Glob Health. 2021 May 22;11:03070. doi: 10.7189/jogh.11.03070 (PMC8219344; doi:10.7189/jogh.11.03070)
Supplement: Online Supplementary Document [file jogh-11-03070-s001.pdf]

Supplementary Data

Supplementary Table 1

Quality of experience between an in-person conference (CamWHO 2019) versus and online conference (CamWHO 2020), as reported by participants.

An identical questionnaire, in line with GDPR regulations, was used after both conferences to allow for more direct comparison of the effects and experiences of the events, as reported by participants. Participants were asked to rate their experiences on a scale from 1-10 with 1 representing poor and 10 representing excellent understanding/confidence.

	2019 (in-person) (n=37)			2020 (online) (n=45)		
	Before CamWHO	After CamWHO	Difference	Before CamWHO	After CamWHO	Difference
Understanding of the WHO	5.2	7.6	+2.4	5.1	7.9	+2.8
Understanding of Global Health	6.3	7.8	+1.5	6.5	8.2	+1.7
Understanding of the theme	5.8	8	+2.2	5.4	7.9	+2.5
Confidence in speaking and debating	5.5	6.8	+1.3	5.5	7.3	+1.8
